# Implementation strategies, and barriers and facilitators for implementation of physical activity at work: a scoping review

**DOI:** 10.1186/s12998-019-0268-5

**Published:** 2019-10-09

**Authors:** Anne Garne-Dalgaard, Stephanie Mann, Thomas Viskum Gjelstrup Bredahl, Mette Jensen Stochkendahl

**Affiliations:** 1 Department of Sports Science and Clinical Biomechanics, University of Southern Denmark, Odense, Denmark; 2 Nordic Institute of Chiropractic and Clinical Biomechanics, Odense, Denmark

**Keywords:** Implementation, Physical activity, Workplaces, Theoretical Domains Framework, Barriers, Facilitators, Scoping review

## Abstract

**Background:**

Inactivity and sedentary lifestyle have led experts to recommend an increase in structured, workplace-based physical activity (PA) initiatives. Previous studies on workplace-based PA have only shown moderate and short-term effects. This has been attributed to the lack of clear implementation strategies and understanding of factors that may hinder or enable uptake of PA. To ensure long-term, sustainable outcomes, there is a need for a better understanding of implementation strategies, and barriers and facilitators to workplace-based PA.

**Method:**

A scoping review of studies investigating implementation approaches and factors affecting uptake of workplace-based PA was conducted. Qualitative and quantitative articles published in MEDLINE, Embase, Scopus, or PsycINFO between 2008 and 2018 evaluating the implementation of PA were included. Data on study characteristics, evaluation, and implementation methods applied were systematically extracted. Two reviewers extracted, coded, and organised factors affecting uptake using the Theoretical Domains Framework (TDF).

**Results:**

After dual, blinded screening of titles and abstracts, 16 articles reporting on eight studies were included in the review. Several different methods of implementation were applied, including information meeting, kick-off events, and “change agents” as the most common. A total of 109 factors influencing implementation were identified, consisting of 57 barriers and 52 facilitators. Barriers most often related to the TDF domains *Environmental Context and Resources* (*n* = 34, 36.2%), *Social influences* (*n* = 13, 13.8%), and *Social/Professional Role and Identity* (*n* = 8, 8.5%). Likewise, facilitators most often related to the TDF domains *Social influences* (*n* = 17, 19.5%), *Environmental Context and Resources* (*n* = 16, 18.4%), and *Social/Professional Role and Identity* (*n* = 9, 10.3%).

**Conclusion:**

Our review has highlighted the multilevel factors affecting the uptake of workplace-based PA and underpins the complexities in implementation of such initiatives. The published literature predominantly provides details from the employees’ perspectives on factors that need to be addressed and a lack of attention to these factors will cause them to hamper uptake of PA. The analysis of barriers and facilitators provides a theoretical foundation to guide future intervention design. However, further research is needed to fully understand the success or failure of implementation processes.

**Electronic supplementary material:**

The online version of this article (10.1186/s12998-019-0268-5) contains supplementary material, which is available to authorized users.

## Background

Physical activity (PA) has been considered as health enhancing for decades, with health authorities advising that PA should be part of daily life throughout the lifespan. Most of the adult working population spends a great deal of their time at work, and general changes in working environments have increased time spent on sedentary work [1]. Inactivity and sedentary lifestyles have been estimated to cause over 1 million deaths annually in Europe [2], which have led experts to recommend an increase in structured PA embedded in modern work activities [3]. This has changed the focus on workplaces from being an arena for work-related activities only, to also becoming a potential field for implementing PA and other types of health promoting initiatives.

To mirror this shift in focus, recent studies have evaluated the effect of a variety of PA programmes in the workplace. Positive, moderate, short-term health enhancing benefits have been documented for the cardiovascular system [4–6], the metabolism [5, 6], musculoskeletal pain and function [7–11], and mental health and general wellbeing [12, 13]. In addition, moderate evidence also points towards a positive effect of workplace health promotion on work ability [4, 12, 13].

When it comes to the impact of workplace PA on other types of work performance outcomes, like productivity [14, 15], presenteeism [16], and work absence [12, 13], the evidence is sparser and the results inconsistent [14–16]. Recent randomised controlled trials (RCT) have not provided conclusive evidence of the effect on work performance outcomes [4, 5, 7], but indicate that attending PA during working hours does, at least, not appear to have negative effects on work productivity [4, 5].

Workplace-based PA encompasses a wide range of activities of various duration, intensity, and mode of delivery. But a common feature is the targeted nature of these activities to the needs of the individuals beyond general advice about health enhancing behaviors. In 2015, Pereira et al. identified eight workplace-based RCTs, which encompassed such diverse PA programs as strength training, aerobic training, combinations of the two, activities targeting flexibility, different types of walking programs, yoga, and tai chi [14]. In Denmark, no less than 15 RCTs have been conducted, which have tested the concept of intelligent physical exercise training (IPET) [5]. IPET is delivered as individually tailored PA to match the individual’s work exposure, health status, and physical capacity, and it includes aerobic training, strength training targeting e.g., neck and shoulder muscles, core stability training, and balance training [5]. IPET has been tested in various working groups with varying effects [5]. Improvements in musculoskeletal pain were mostly found among office workers, dentists, industrial laboratory technicians, cleaners, and fighter pilots, whereas improvements in the cardio-metabolic systems were mostly seen among office workers, healthcare workers, and construction workers [5].

When positive, the effects of PA appear to be only modest and short-term. One reason being that implementation and adherence to workplace PA has proven difficult [17–19]. As an example, the mean level of regular adherence was 61% across nine RCTs conducted in Denmark, ranging from 31% to 86% [17]. Baily et al. systematically reviewed barriers and facilitators for implementation of workplace physical activity policies and found that not having a clear company policy on workplace PA is a vital barrier for implementation [20]. Other researchers have suggested that the suboptimal results of implementation of general health enhancing interventions could be due to limited use of a theoretical foundation to underpin such interventions [21, 22]. Several implementation theories, models, and frameworks exist, which may provide better understanding and explanation of how and why implementation succeeds or fails [21]. Nilsen suggests the use of determinant frameworks to describe factors that impact implementation outcomes, i.e., factors that either impede or enable uptake [21]. One of these determinant frameworks is the widely used Theoretical Domains Framework (TDF). TDF is a comprehensive synthesis of theories of behaviour and behaviour change [23, 24]. It is a multilevel framework, which allows identification of determinants at different levels, from the individual user, to the organization and beyond [21]. Like many other determinant frameworks, it does not specify causal mechanisms, but provides potentially useful information for designing and executing implementation strategies [21].

Despite comprehensive research on PA in workplaces, the implementation and sustainability of such initiatives are still challenging and need more attention [9, 16, 17]. During the last 30 years, there has been important development in the content, performance, and organization of work in many industries. At the same time, significant changes in workers’ health have occurred. In 2009, a systematic review by Robroek et al. [25] investigated determinants of participation in general worksite health promotion programs. The interventions consisted of education or counselling as main component, introductions of a fitness center or exercise facilities, or multi-component programs. The authors evaluated determinants of participation at individual, workplace and intervention type levels. They found large variations in participation levels, and only female sex was associated with higher participation in the pooled analyses. This led Robroek et al. to conclude: *“Few studies evaluated the influence of health, lifestyle and work-related factors on participation, which hampers the insight in the underlying determinants of initial participation in worksite health promotion*” [25]. To ensure long-term, sustainable implementation of workplace-based PA, there is a need for a better understanding of implementation strategies, and the barriers and facilitators that impact uptake of workplace PA interventions. A greater insight into these factors would enhance the opportunity to more accurately tailor interventions and thereby, increase the opportunity of successful implementation and long-lasting effects on employees’ health and well-being, and workplace performance outcomes.

Therefore, the aim of this scoping review is to descriptively summarize implementation approaches for workplace-based PA, and to identify and organise barriers and facilitators affecting the uptake of the workplace-based PA using TDF.

## Method

### Study design

This scoping review is reported according to the Preferred Reporting Items for Systematic Reviews and Meta-Analyses Extension for Scoping Reviews (PRISMA-ScR) [26].

### Information sources and search strategy

A systematic search of the following databases was undertaken: Embase, MEDLINE, Scopus, and PsycINFO. All databases were searched from 2008 until March 2018. Reference and citation searching were also undertaken. The searches were performed by the first author, who was guided by an experienced information specialist from the library at the University of Southern Denmark.

The search strategy included subject indexing terms and free-text terms for title, abstract, and keyword searching. The research question directed the entire literature search, and based on this, the search terms were grouped into three concepts and arranged in accordance to relevance in a search matrix: 1) implementation, 2) physical activity, and 3) workplaces. Search terms under each of the three concepts were selected from keywords identified in a preliminary search in PubMed and the list of Medical Subject Headings (MeSH), and after discussion with the review team. The full version of the search terms used, including specifications on use of title, keywords, or abstract screening, is documented in Additional file 1. In all four databases, the search was performed according to the block search method using the Boolean operators “OR” and “AND”. To maintain an overview throughout the literature search, a search protocol was continually populated with all search terms, number of hits, and combination of searches (Additional file 1).

### Study selection

All identified citations from the searched databases were uploaded to EndNote ×8 software. An integrated duplication detection tool was used to identify duplicates. All suggested duplicate pairs were screened for correctness by one reviewer (AGD). Title and abstract screenings were performed for each article by two independent reviewers (AGD and SM) [27]. Disagreement between the two reviewers resulted in inclusion of the citation to full-text screening. Full-text screening was similarly performed by two independent reviewers (AGD and SM) assessing the eligibility of the citation. Any disagreements were resolved through discussion mediated by a third reviewer (MJS) [27].

The eligibility criteria for the original studies are presented in Table 1. Unlike systematic reviews and meta-analyses, the eligibility does not necessarily have to be established before the literature search in a scoping review. These can be developed as the knowledge of the identified literature grows [29]. In accordance with this approach, the eligibility criteria for this scoping review were adjusted after the screening of the titles. Interventions relating to return-to-work, sickness absence management, and occupational safety were added to the exclusion criteria, due to irrelevance in accordance with the review focus.

**Table 1:** Inclusion and exclusion criteria

Inclusion criteria	Exclusion criteria
Participants: age 18+, employed	Participants: age under 18, unemployed
Content:	Content:
Intervention: Implementation of physical activity at workplaces:	Intervention: Implementation of other kinds of “Employee health”:
Physical training	Ergonomics
Physical exercises	Active transport to/from work
Exercise	Promotion of health, e.g., via e-mail
“Active breaks”	Diet
Flexibility and mobility exercises	Smoking
Outcome:	Alcohol
Any kind of evaluation of the implementation process. Qualitative, quantitative, or mixed methods	Psychological work environment
	“Return-to-work” interventions
	“Sickness absence management”
	Occupational safety
	Walking/walking on stairs
	Outcome:
	No evaluation of the implementation process
Context: Any kind of workplace	Context: Other than workplaces
Type of publication: All types	
Year of publication: After 2008	
Languages: English, Danish, Swedish, Norwegian	

The development of the inclusion and exclusion criteria was guided by Joanna Briggs Institute: Reviewer’s Manual, chapter 11.2.4 on inclusion criteria in scoping reviews [28]

### Data collection

Similar to the study selection process, data extraction was performed independently by two reviewers (AGD and SM) using predefined data extraction spreadsheets. Discrepancies in data extracted were negotiated until consensus was reached. Data were systematically extracted on study characteristics (year, country, study design); study participants (occupation, number of participants); intervention (content, duration); implementation approaches and evaluation (details on methods of implementation, evaluation methods applied, main findings); and barriers and facilitators (methods of data extraction, factors or themes of either enhancing or hampering effect on implementation). In qualitative studies, barriers and facilitators were extracted in their original format, unless the authors had coded the factors to specific themes indicating otherwise. In quantitative studies, factors were extracted if 50% or more of the participants indicated the factor as a barrier or facilitator. If the authors of the original study did not state the factors as barriers or facilitators, the reviewers would assess the influence on the implementation, as either enhancing/positive (facilitator) or hampering/negative (barrier).

### Sorting the data using the theoretical domains framework

To further organize and make sense of the data, we used TDF to code the extracted barriers and facilitators into domains. TDF contains 14 domains, which offers a theoretical perspective on the cognitive, affective, social, and environmental influences on behaviour [24, 30].

A coding manual (Additional file 2) was developed to guide the coding process [23, 30, 31]. Two coders (AGD and SM) independently coded barriers and facilitators. Barriers and facilitators could be coded to more than one domain if deemed relevant. Any disputes about the meaning of domain definitions or coding of factors were resolved by discussion until agreement was reached.

### Data synthesis

The study population, implementation strategies, evaluative methods and key findings of the included studies were descriptively summarised and presented. The results of the TDF coding process were summarised for each domain, and the absolute number and proportion of codes was calculated for the barriers and facilitators respectively. Lastly, the main themes from the coding process were identified and examples from identified barriers and facilitators were presented to illustrate the coding process.

## Results

### Study selection

**Fig. 1:**
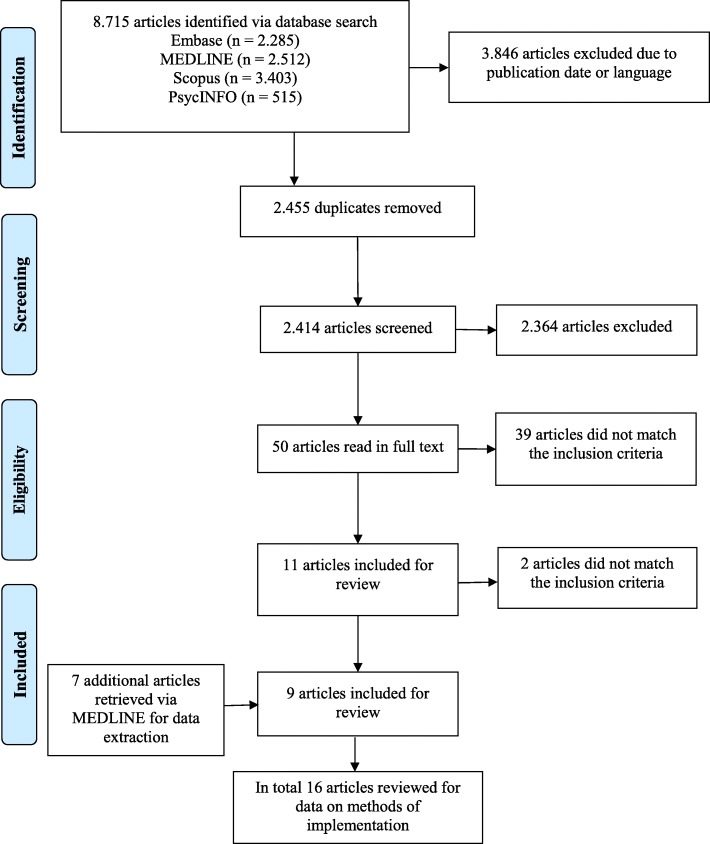
PRISMA Flowchart

We identified a total of 8,715 citations. From these, 2,455 citations were excluded as duplicates and 3,846 citations were excluded due to publication date or language. A total of 2,414 titles and abstracts were screened, which resulted in screening of 50 full-text papers. Nine references met the inclusion criteria [32–40]. Additionally, seven references were identified by searching reference lists of included studies [17, 41–46]. This resulted in a total of 16 included articles concerning eight different studies. The PRISMA flow diagram demonstrating the selection process is illustrated in Fig. 1 [47].

### Description of included studies

Table 2 presents an overview of the included studies. Three of the eight studies were undertaken in the United States [32–34, 39], three in Denmark [35, 38, 40], and two in the United Kingdom [36, 37]. The studies were published between 2013 and 2018. Four studies were qualitative [32, 33, 36, 39, 40], one study was quantitative [38], and three studies were mixed-methods [34, 35, 37]. The reports included a total of 2,686 participants with a range of 41–1,260 participants per study.

**Table 2:** Overview of included studies and articles

#	Authors, year, country	Design	Participants	Intervention	Methods of implementation	Methods of evaluation	Authors’ main conclusions	Comments
#1	Taylor et al. 2013, USA Taylor et al. 2014, USA Taylor et al. 2010, USA	Qualitative and quantitative	Office and hospital employees (*n* = 82)	Booster breaks. Cardio, strength, relaxation exercises in groups of 5–15 participants. 15 min. Per day for 6 or 12 months. In lunch or conference rooms at 5 worksites.	Kick-off event, “ambassadors” partnership (Break Buddies), prizes and economic incentives, definite schedule for exercise sessions, hand out manuals.	Interviews with 24 participants. Survey: Three open-ended questions on the interventions effect on the lives of the participants and suggestions for improvements and a story path method – before, during, after the intervention	Advantages: Positive feelings and reduced stress, enhanced focus on health, strengthened social interactions and organisational support. Barriers: Lack of time, motivation and social/organisational support and no variation in the training	Only the most frequently occurring themes are mentioned
#2	Tudor-Locke et al. 2014, USA	Mixed methods	Office employees (*n* = 41)	The WorkStation Pilot Study. Treadmill workstations. Individually, scheduled rotation among workstations. Two daily sessions of 45 min. For 6 months	Kick-off event, prizes and economic incentives, follow-up from the research team, optional support via phone or email	Web-based, post-session surveys and focus group interviews	Most common reasons for absence were conflict with work tasks, not in the office or sickness absence. Most of the participants were positive toward treadmill workstations.	Very low participation rate. 17% responded to recruitment. 5.6% attended baseline.
#3	Justesen et al. 2017, DK Sjøgaard et al. 2014, DK	Mixed methods	Office employees (*n* = 389)	IPET ^a^ -Individually tailored cardio, strength, functional exercise. 1 h per week for 2 years. At worksites or in the local area.	Information meetings for the participants, “change agents”, instructors/supervision, handout manuals, individually adjusted training, log books/training journals	Survey questions for all employees, fieldnotes from meetings with change agents, survey and focus group interview with change agents, and survey and interview with middle management	Middle management plays a major role in the implementation of physical exercise. But they are often unsure about this role and tend to leave all responsibility to the top management	Only the middle manager’s role in the implementation process is evaluated.
#4	Kinnafik et al. 2018, UK Shepherd et al. 2015, UK	Qualitative and quantitative	University employees (*n* = 46)	Indoor bicycles HIIT ^b^ program. In groups. 18–25 min. 3 times per week for 10 weeks. In close proximity to the worksite.	Information meetings for the participants, flexible schedule for exercise sessions, instructors/supervision, individually adjusted training	Focus group interviews guided by the RE-AIM framework and observation notes from focus group interviews	HIIT is an acceptable and efficient method of exercise for employees who are insufficiently physically active. Social factors influenced the level of adherence. Despite this, participants were reluctant to continue with the HIIT training.	12 participants in the evaluation
#5	Lawton et al. 2014, UK McEachan et al. 2011, UK	Mixed methods	Employees at a bus company, hospital, university, city council, and government agency (*n* = 1260)	“AME for Activity” (Awareness, Motivation, Environment). A toolkit of activities to increase physical activity. Team based. 3 months. 44 worksites.	Kick-off event, “ambassadors” focus on visual design, flexible schedule for exercise sessions, hand out manuals, follow-up from the research team	Survey, focus group interview and fieldnotes	The intervention is efficient under ideal circumstances, which entail commitment by facilitators, susceptibility and engagement by employees, and the physical surroundings	
#6	Andersen & Zebis 2014, DK Andersen et al. 2011, DK	Quantitative	Office employees (*n* = 198)	To reduce musculoskeletal disorders. Strength training with elastic bands. Individually. 1 exercise for either 2 or 12 min. Daily for 10 weeks. At office worksites.	Information meetings for the participants, instructors/supervision, hand out manuals, planned progression, log books/ training journals, optional support via phone or email	Definitions of reach, dose delivered, dose received, fidelity and satisfaction. Surveys and training journals	The strength training was generally well accepted by the participants, but more variation is needed. Lack of time and difficulties following illness were the greatest barriers	116 participants in the evaluation
#7	Mayer et al. 2013, USA Mayer et al. 2013, USA	Qualitative and quantitative	Firefighters, fulltime (n = 94)	Supervised exercise. Strengthening of back and core muscles. 2 sessions of 10–15 min. Per week for 24 weeks. At fire stations.	Flexible schedule for exercise sessions, instructors/supervision, planned progression	Focus group interviews. Three with employees and one with leaders. Eight open-ended questions	Lack of self-motivation, support from colleagues and time during the workday were the greatest barriers	27 participants in the evaluation
#8	Bredahl et al. 2014, DK Andersen et al. 2010, DK	Qualitative	Office employees (*n* = 573)	VIMS ^c^. Five strength training exercises with dumbbells for shoulders, neck and arms. 1 h per week for 20 weeks. At the worksites or in the local area.	Group exercise to enhance social relationships, focus on visual design, definite schedule for exercise sessions, instructors/supervision, planned progression, log books/ training journals, optional support via phone or email	Semi-deductive, structured thematical interviews. Three themes: organisation, implementation, and individual	The greatest barrier for participation was the internal working culture. A clear connection between management’s intentions and an actual implementation is crucial. Furthermore, it is important to structure the intervention and secure flexibility during the working hours, to enable employees to participate.	18 participants in the evaluation

^a^*IPET* Intelligent Physical Exercise Training, ^b^ *HIIT* High Intensity Interval Training, ^c^ *VIMS* company adjusted intelligent exercise for pain in neck and shoulders

### Study population

Five out of eight studies included office workers and/or white-collar workers with mostly sedentary work [32–35, 38, 40]. One study included university employees [36], and one study included participants from five different companies (bus company, hospital, university, city council, and government agency) [37]. One study focused on employees who did not meet the recommendations for physical activity [36], and one study included employees who were experiencing pain in the neck and shoulders [40]. Only one study focused on an occupation with high physical work demands (firefighters) [39].

### Interventions

In five out of eight included studies the physical activity intervention was comprised by cardio [32, 33, 35], strength [32, 33, 35, 38–40] or functional exercises [35]. The remaining three interventions encompassed treadmill workstations [34], an indoor bicycle HIIT program [36] and a motivating toolkit and team challenges [37]. Three interventions were group-based [32, 33, 36, 37] and five focussed on the individual [34, 35, 38–40]. The interventions either took place at the worksite [32, 33, 38, 39], near the worksite [36] or both [35, 37, 40]. The duration of the interventions spanned from 10 weeks to 2 years and incorporated different time schedules. E.g. Taylor et al. applied exercises 15 min a day, one study applied indoor bicycle HIIT for 18–25 min, three times a week [36] and another scheduled exercise for 1 hour a week [35]. Also, different methods of instruction were applied across the interventions, differing from trained participants being in charge of instruction [32, 33], to professional instructors overseeing some [35, 38, 40] or all training sessions [36, 39].

### Methods of evaluation

Five out of eight included studies applied more than one method of evaluating the implementation process [34–38]. The most commonly applied methods were focus group interviews (*n* = 5) [34–37, 39] and surveys (n = 5) [33–35, 37, 38]. Individual interviews were used in three studies [32, 35, 40], fieldnotes in three studies [35–37], and one study used training diaries to evaluate implementation [38]. Six out of eight studies evaluated the implementation from the employees’ perspectives only [32–34, 36–38, 40], and two studies evaluated from the managers’ and employees’ perspectives [35, 39].

### Implementation strategies and facilitation of intervention delivery

The interventions were initiated by information meetings for the participants in three studies [17, 38, 42, 44], and by a kick-off event in three other studies [34, 41, 43]. Three studies used handpicked employees, who were given between one and three days of instruction, as “change agents” or “ambassadors” to facilitate implementation [17, 35, 37, 41, 43]. Other methods of implementations were group exercise to enhance social relationships [46], and partnership through signed declarations of support (Break Buddies) [41]. Two studies offered prizes and economic incentives for participation in the intervention [34, 41]. Only two studies described the visual design of the intervention with regards to information material, logos, and posters [43, 46]. Bredahl et al., 2015, described a focus on the physical surroundings, in terms of light and friendly training environment and colourful posters on the walls showing the training exercises [46].

### Theoretical domains framework - barriers and facilitators

A total of 109 factors were identified in the eight included studies and were divided between 57 barriers and 52 facilitators. When applying the TDF, the 109 factors were given 181 codes: 94 codes to the barriers and 87 codes to the facilitators. Thus, 53 factors were given codes from more than one TDF domain. Table 3 presents the overall results of the TDF coding. For each study, the number of identified barriers ranged from two to 12, and for facilitators from three to nine. The barriers were coded under 11 of the 14 TDF domains and most frequently, to the TDF domains *Environmental Context and Resources* (ECR, *n* = 34, 36.2%), *Social influences* (*n* = 13, 13.8%), and *Social/Professional Role and Identity* (SPRI, *n* = 8, 8.5%). We did not find barriers relating to the domains *Optimism, Reinforcement* and *Memory, Attention and Decision Processes*. Likewise, the facilitators were most frequently coded to the TDF domains *Social influences* (*n* = 17, 19.5%), *Environmental Context and Resources* (ECR, *n* = 16, 18.4%), and *Social/Professional Role and Identity* (SPRI, *n* = 9, 10.3%). The facilitators covered all 14 TDF domains. For an overview of the TDF coding, see Additional file 3.

**Table 3:** Overall results of the TDF coding process

TDF Barriers (*n* = 94)			TDF Facilitators (*n* = 87)		
Domain	(n)	(%)	Domain	(n)	(%)
ECR	34	36.2%	Social influences	17	19.5%
Social influences	13	13.8%	ECR	16	18.4%
SPRI	8	8.5%	SPRI	9	10.3%
Skills	7	7.4%	Beliefs about capabilities	8	9.2%
Beliefs about consequences	7	7.4%	Goals	8	9.2%
Intentions	7	7.4%	Knowledge	6	6.9%
Beliefs about capabilities	6	6.4%	Emotion	6	6.9%
Emotion	5	5.3%	Behavioural regulation	5	5.7%
Knowledge	4	4.3%	Skills	4	4.6%
Goals	1	1.1%	Intentions	3	3.4%
Behavioural regulation	2	2.1%	Reinforcement	2	2.3%
Optimism	0	0%	Beliefs about consequences	1	1.1%
Reinforcement	0	0%	Optimism	1	1.1%
MADP	0	0%	MADP	1	1.1%

*TDF* Theoretical Domains Framework, *SPRI* Social/professional role and Identity, *ECR* Environmental Context and Resources, *MADP* Memory, Attention and Decision Processes

Barriers and facilitators coded to the *ECR* domain covered organisational culture and resources and employees’ interactions with, and influences of, the surrounding environment. Under the ECR domain, all eight studies identified lack of time, conflicting work time schedules, or alternating workstations/work locations as barriers [32, 34–40]. E.g., in Taylor et al., 48% of the data texts collected from interviews regarding barriers concerned issues with scheduling the exercise, time constraints, and interruptions of work flow [32]. Further, vacation or sickness absence interrupting the program was perceived as a barrier [34, 37]. Several studies reported suboptimal implementation because of a lack of awareness among employees about the intervention [36, 37] or workstations not suitably equipped for both work and exercise [34]. For example, Tudor-Lock et al. reported how fitting a rigid schedule of shared use of treadmill desks with the equally rigid work schedule was challenging [34]. In addition, the treadmill desks lacked necessary equipment and compromised the confidentiality during telephone calls [34]. Four studies reported that study participants lost motivation because the exercise programs lacked variation [33, 38–40], and in one study, some participants were disinclined to engage in workplace-based exercise, as they felt this activity belonged to leisure time and private life rather than work life [35]. Other barriers coded under the *ECR* domain were disturbing noises from workstation treadmills [34] and increased room temperature as a result of exercising [36].

Facilitators under the ECR codes related to the positive evaluations of the content of the exercise programs with respect to intensity and frequency of the exercises [36, 38], the simplicity of the program [39], and the flexibility of the programs in terms of location, time spent on exercising, and timing during the workday [36, 40]. In two studies, the opportunity to meet and spend time with colleagues was mentioned as a facilitating factor [37, 40]. Further, structured breaks [32], change agents [35], the work team working in the same location [37], and clear distribution of roles and responsibilities between project implementers and management [35] were reported as facilitating.

Factors that impact uptake of the interventions and coded to the *Social influences* domain covered interpersonal processes, social support and norms, group conformity and identity, and intergroup conflict. Three studies reported lack of managerial support [32, 33, 35, 37], and four studies reported lack of social/collegial support as factors which impede uptake [32, 36, 39, 40]. For example, lack of camaraderie amongst colleagues was reported as a barrier by Taylor et al. [32], and in Mayer et al., participants reported that they would have been more motivated by group-based exercise than the implemented individual exercise [39]. Bredahl et al. reported that doing exercise and sweating in a public place was perceived as a barrier for some participants. In the same study, colleagues were found to hinder PA if they were pressuring others to keep working instead of doing exercise [40]. Other barriers under the *Social influences* domain were lack of motivation/commitment [32, 37] and instructors without appropriate competences and/or behaviour [36, 40].

Just as the lack of social/collegial support was found to impede PA, the presence of this support was reported to facilitate workplace PA [36–40]. Building a sense of team spirit or camaraderie amongst colleagues and doing activities together with colleagues were reported as means to create this support [36, 39, 40]. Further, supervision of the exercise by instructors [36, 38, 39] and support, acknowledgement, and active participation from management facilitated uptake [32, 35, 37, 40].

Factors coded to the *SPRI* domain covered behaviours and personal qualities, professional, social and group identity, and organisational commitment and leadership. Barriers coded to *SPRI* related to inconsistent or lack of support from management [33, 35, 37]. Lawton et al. reported that despite being supportive at the beginning, management would not allow employees the time and resources needed for the intervention [37]. In Taylor et al., participants suggested that management should participate more in the exercise sessions and be more encouraging [33]. Further, lack of project management and unclear roles between implementers/project managers and workplace managers impeded uptake [35].

The *SPRI* facilitators included sense of acceptance and legitimacy of participation as a result of managerial awareness, attitude and support [33, 35, 37, 40]. Other facilitators included team spirit and improved social work atmosphere [32].

Together, the *ECR, Social influences* and *SPRI* codes make up 58.5% of the coded barriers and 48.2% of the coded facilitators. The remaining 41.5% of the barriers are divided between eight domains, with the domains *Skills, Beliefs about consequences, Intentions* and *Beliefs about capabilities* with the highest representation. For the barriers, these domains cover lack of knowledge regarding health promotion, the intervention being too difficult or too easy [34, 35, 39], a sense of interference with one’s private life, the timing of the intervention [35, 37, 39], prioritisation of tasks, lack of commitment [35, 37], difficulties in the execution of the intervention, or loss of motivation in the event of negative results [34, 36]. The remaining 51.8% of the facilitators are divided between the remaining 11 domains, with the domains *Beliefs about capabilities,* and *Goals, Knowledge* and *Emotions* being the most prevalent. Acting as facilitating factors were feelings of enhanced competence and self-confidence [36, 39], or motivation to behaviour change and exercise [33, 40], increased health [32, 34, 39], and decreased pain [39, 40], or the experience of positive feelings towards, and greater joy with, physical activity [32, 34, 36].

## Discussion

### Principal findings

We have systematically searched and descriptively summarized the literature pertaining to implementation approaches of workplace-based PA interventions, and the barriers and facilitators affecting uptake of such interventions. We found a large degree of variety regarding intervention content, delivery, and implementation; thereby, making comparisons between interventions difficult. We applied TDF to organise and describe barriers and facilitators, and found factors related to all 14 TDF domains, which underpins the multilevel dimensions and complexity of implementation of workplace-based PA interventions.

The majority of factors affecting uptake of workplace-based PA were coded to three TDF domains, *ECR*, *Social influences*, and *SPRI*. In most cases, we consistently found that absence of these factors hindered uptake, whereas presence facilitated uptake of the intervention. This indicates that focus and action must be directed toward these factors, otherwise they will hinder the uptake when implementing PA in workplaces. We found both intrinsic factors in the individual participants, like unwillingness to participate in workplace PA as this was believed to infringe on private matters, or participants feeling exposed when exercising in front of colleagues, and extrinsic factors related to different levels in the organisation and the persons acting within the organisation. These included organisation of work task and work schedules, prioritisation and allocation of resources, managerial support and commitment, and the importance of social coherence and group dynamics. The content, flexibility, variation of the intervention and competency of those delivering the intervention also impact uptake.

### Factors affecting uptake of workplace-based PA

We found that a variety of methods have been applied in the implementation of workplace-based PA interventions, but in general, the engagement strategies used were not described in detail. Information meetings, kick-off events, and the application of handpicked, educated employees as “change agents” or “ambassadors” were the most common methods of implementation. The concept of “change agents” is advocated by several theories and models of behaviour change and implementation, and is based on the theory that any process of change needs someone to take the lead [48, 49]. “Change agents” have been found useful in diverse settings and conditions, such as prevention and health promotion programs among children [50]; weight management in adults with intellectual disabilities [51]; application of ergonomics among industrial workers [52]; and resident-oriented care in nursing homes [53]. We found that individually tailored interventions, adaptability, and flexibility in intervention programs increase uptake. This finding is supported by reviews of both workplace-based health programs [16], and in implementation studies from a wide range of health conditions and situations [50]. Other authors have advocated for short rather than long exercise programs [15]. Further, in concordance with other workplace-based health programs, which have shown that various incentives (e.g., gifts and gift vouchers) improve participation and intervention adherence [16], we found that offering financial incentives increases uptake. Also in line with our results, several other studies stress the importance of leadership support, a collective sense of ownership, allowing employees to take part in the development, a supportive workplace culture, and focus on economic advantages, when implementing health promoting interventions [16, 20, 50, 54]. Identifying which engagement and recruitment strategies are most effective for different groups of workers would be beneficial. Conceptual frameworks and metrics have been developed in other areas of health research to help determine which approaches are most successful [55, 56]. It will be important that future studies describe engagement or recruitment strategies in greater detail to improve the fidelity and impact of these approaches using resources and guides like the template for intervention description and replication (TIDieR) checklist [56, 57].

The majority of identified barriers and facilitators were coded to the TDF domains *ECR, Social influences,* and *SPRI*. This mirrors, to some extent, the results of other studies, which likewise found *ECR* and *Social influences* to be of greatest influence when implementing PA. This has been seen in implementation of PA in school settings [31, 58], and in mental and physical rehabilitation settings [59–61]. Some factors (e.g., interaction with colleagues) were perceived differently among participants, and thus reported as both a barrier and a facilitator. This was observed both within studies and across studies, which further emphasizes the complexity of implementing PA and the importance of attention to individuality and context when designing interventions. Nearly all the included studies exclusively examined company employees’ perspectives on barriers and facilitators for implementation of workplace-based PA. Since successful implementation processes of health promoting interventions seem to rely on leadership support and a collective contribution [16, 20, 54], research of factors influencing other stakeholders, e.g., management or intervention deliverers could add to the understanding of success or failure of implementation efforts.

### Strengths and limitations

This scoping review was designed and reported in line with the recommendations of the PRISMA-ScR statement [26]. We searched multiple databases, and a thorough search strategy was designed iteratively by the research team, and an information specialist, to account for the three different dimensions of the search (implementation, physical activity, and workplaces). All aspects of data collection, data extraction, and data analysis were carried out independently by two researchers, with a third party available for mediation in case of disagreements. We applied TDF to provide a structured and systematic foundation on which implementers may base the design of future interventions [62]. The application of TDF provides a theoretical foundation for working with implementation and factors affecting uptake, and gives a comprehensive investigation of potential factors relating to implementational difficulties [23]. The primary limitation of this scoping review is the sparse literature related to our objectives. Due to the limited number and type of study designs of the original studies, and the purpose of our review, we were not able to elucidate any relationship between factors that impact uptake of physical activity and the population or the content of the intervention.

Additionally, our search was limited to studies published from 2009 and onwards, and in English, Danish, Swedish, or Norwegian. The restrictions in language could be taken as a limitation, although there is some indication that this has only marginal impact on results [63], and grey literature was not included. We did not assess the quality of the included studies, as this is usually not part of a scoping review, due to an effort to keep a wide perspective and include studies with different methods and designs [29].

### Future research and practical application

We have found many different factors affecting the success of implementation of workplace-based PA interventions, which highlight the need for well-planned implementation processes considering multiple levels and factors. In workplace-based PA studies, only few have undertaken process evaluations and even fewer base these on a theoretical framework [64]. This is a well-known challenge in implementation science, and studies addressing these issues, have been sought by researchers in the fields of implementation and health promotion [21, 22, 64]. Future research should focus on the application of theoretically informed process evaluations including all stakeholders, to provide standardised information on successful and unsuccessful implementation methods. We recommend implementers of workplace-based PA to apply a systematic approach, which ensures consideration of all influencing factors. Incorporation of a thorough assessment of needs and available resources and involvement of all participating parties would likely assist the process positively. Using implementation frameworks, like TDF, the linking of theories of behaviour change to behaviour change techniques and approaches becomes more tangible and relevant [30], thus enabling a better connection between intervention functions and behaviour change techniques to further guide intervention design [65].

## Conclusions

Our review has highlighted the multilevel factors affecting the uptake of workplace-based PA and it underpins the complexities in implementation of such initiatives. The published literature predominantly provides details from the employees’ perspectives on factors that need to be addressed, and a lack of attention to these factors could cause them to hamper uptake of PA. The analysis of barriers and facilitators provides a theoretical foundation to guide future intervention design. However, it is clear that further research is needed to fully understand the success or failure of implementation processes.

## Additional files

Additional file 1: Search matrices and protocols. Search matrix + protocol for each of the four databases. (DOCX 21 kb)

Additional file 2: TDF coding manual. TDF domains, definitions and constructs used to code barriers and facilitators. (DOCX 18 kb)

Additional file 3: Overview of the TDF coding. Total number of identified factors and results of TDF coding for the included studies. (DOCX 17 kb)

## Data Availability

All data generated or analysed during this study are included in this published article [and its supplementary information files].

## Abbreviations

ECR: Environmental Context and Resources
HIIT: High Intensity Interval Training
IPET: Intelligent Physical Exercise Training
MADP: Memory, Attention, and Decision Processes
MeSH: Medical Subject Headings
PA: Physical activity
PRISMA: Preferred Reporting Items for Systematic reviews and Meta-Analysis
PRISMA-ScR: Preferred Reporting Items for Systematic reviews and Meta-Analysis extension for Scoping Reviews
RCT: Randomised controlled trial
SPRI: Social/Professional Role and Identity
TDF: Theoretical Domains Framework
