# Supplementary material for: Implementation strategies, and barriers and facilitators for implementation of physical activity at work: a scoping review
Source: Chiropr Man Therap. 2019 Oct 9;27:48. doi: 10.1186/s12998-019-0268-5 (PMC6784342; doi:10.1186/s12998-019-0268-5)
Supplement: Supplementary file 1 — Search matrices and protocols. Search matrix + protocol for each of the four databases. (DOCX 21 kb) [file 12998_2019_268_MOESM1_ESM.docx]

Additional file 1 (.docx). Search matrices and protocols. Search matrix + protocol for each of the four databases.

MEDLINE search matrix

| MEDLINE (PubMed) | | |
| --- | --- | --- |
| 1: Implementation | 2: Physical activity | 3: Workplaces |
| Implement*  Execution  Compliance [TiAb]  Adherence  Treatment adherence  and compliance [MeSH]  Adaptation [TiAb]  Adaptation, psychological [MeSH] | “Physical activity”  “Physical exercise”  “Physical training”  Exercise  Exercise [MeSH]  “Workplace intervention”  “Occupational health”  Occupational health [MeSH] | “Work place”  “Work site”  “Work station”  Workplace  Workplace [MeSH]  Worksite  Workstation  “Work location”  “Job site” |

MEDLINE search protocol

| MEDLINE (PubMed) | | Date: 20/3 2018 |
| --- | --- | --- |
| **Nr. #** | **Search terms** | **Number of hits** |
| 1 | Implement* | 387,776 |
| 2 | Execution | 20,815 |
| 3 | Compliance [TiAb] | 101,580 |
| 4 | Adherence | 128,909 |
| 5 | Treatment Adherence and Compliance [MeSH] | 211,457 |
| 6 | Adaptation [TiAb] | 141,637 |
| 7 | Adaptation [MeSH] | 116,323 |
| 8 | 1 or 2 or 3 or 4 or 5 or 6 or 7 | 1,317,047 |
| 9 | “Physical activity” | 91,800 |
| 10 | “Physical exercise” | 13,160 |
| 11 | “Physical training” | 5,369 |
| 12 | Exercise | 366,641 |
| 13 | Exercise [MeSH] | 162,054 |
| 14 | “Workplace intervention” | 221 |
| 15 | “Occupational health” | 69,686 |
| 16 | Occupational health [MeSH] | 30,195 |
| 17 | 9 or 10 or 11 or 12 or 13 or 14 or 15 or 16 | 480,848 |
| 18 | “Work place” | 1,858 |
| 19 | “Work site” | 792 |
| 20 | “Work station” | 389 |
| 21 | Workplace | 41,945 |
| 22 | Workplace [MeSH] | 18,751 |
| 23 | Worksite | 43,391 |
| 24 | Workstation | 4,948 |
| 25 | “Work location” | 139 |
| 26 | “Job site” | 73 |
| 27 | 18 or 19 or 20 or 21 or 22 or 23 or 24 or 25 or 26 | 112,286 |
| 28 | 8 and 17 and 27 | 2,512 |

Where nothing is indicated, “All fields” was searched.

Embase search matrix

| Embase | | |
| --- | --- | --- |
| 1: Implementation | 2: Physical activity | 3: Workplaces |
| Implement*  Execution  Compliance  Adherence  Patient compliance [Emtree]  Adaptive behavior [Emtree] | “Physical activity”  Physical activity [Emtree]  “Physical exercise”  “Physical training”  Exercise  Exercise [Emtree]  “Workplace intervention”  “Occupational health”  Occupational health [Emtree] | “Work place”  “Work site”  “Work station”  Workplace  Workplace [Emtree]  Worksite  Workstation  “Work location”  “Place of employment”  “Job site” |

Embase search protocol

| Embase | | Date: 19/3 2018 |
| --- | --- | --- |
| **Nr. #** | **Search terms** | **Number of hits** |
| 1 | Implement* | 487,647 |
| 2 | Execution | 25,366 |
| 3 | Compliance | 275,295 |
| 4 | Adherence | 164,527 |
| 5 | Patient compliance [Emtree] | 119,639 |
| 6 | Adaptive behaviour [Emtree] | 53,410 |
| 7 | 1 or 2 or 3 or 4 or 5 or 6 | 922,073 |
| 8 | “Physical activity” | 116,145 |
| 9 | Physical activity [Emtree] | 124,027 |
| 10 | “Physical exercise” | 18,335 |
| 11 | “Physical training” | 7,508 |
| 12 | Exercise | 430,996 |
| 13 | Exercise [Emtree] | 245,054 |
| 14 | “Workplace intervention” | 265 |
| 15 | “Occupational health” | 57,703 |
| 16 | Occupational health [Emtree) | 38,690 |
| 17 | 8 or 9 or 10 or 11 or 12 or 13 or 14 or 15 or 16 | 600,438 |
| 18 | “Work place” | 2,768 |
| 19 | “Work site” | 901 |
| 20 | “Work station” | 706 |
| 21 | Workplace | 53,092 |
| 22 | Workplace [Emtree] | 33,894 |
| 23 | Worksite | 2,976 |
| 24 | Workstation | 7,870 |
| 25 | “Work location” | 172 |
| 26 | “Place of employment” | 253 |
| 27 | “Job site” | 106 |
| 28 | 18 or 19 or 20 or 21 or 22 or 23 or 24 or 25 or 26 or 27 | 65,933 |
| 29 | 7 and 17 and 28 | 2,285 |

Where nothing is indicated, “All fields” was searched.

Scopus search matrix

| Scopus | | |
| --- | --- | --- |
| 1: Implementation | 2: Physical activity | 3: Workplaces |
| Implement*  Execution  Compliance  Adherence  Adaptation | “Physical activity”  “Physical exercise”  “Physical training”  Exercise  Training  “Workplace intervention”  “Occupational health” | “Work place”  “Work site”  “Work station”  Workplace  Worksite  Workstation  “Work location”  “Place of employment”  Jobsite |

Scopus search protocol

| Scopus | | Date: 20/3 2018 |
| --- | --- | --- |
| **Nr. #** | **Search terms** | **Number of hits** |
| 1 | Implement* | 2,122,290 |
| 2 | Execution | 177,051 |
| 3 | Compliance | 347,874 |
| 4 | Adherence | 178,009 |
| 5 | Adaptation | 562,646 |
| 6 | 1 or 2 or 3 or 4 or 5 | 3,210,562 |
| 7 | “Physical activity” | 161,613 |
| 8 | “Physical exercise” | 20,451 |
| 9 | “Physical training” | 8,199 |
| 10 | Exercise | 541,654 |
| 11 | “Workplace intervention” | 770 |
| 12 | “Occupational health” | 73,859 |
| 13 | 7 or 8 or 9 or 10 or 11 or 12 | 712,891 |
| 14 | “Work place” | 6,570 |
| 15 | “Work site” | 2,620 |
| 16 | “Work station” | 3,652 |
| 17 | Workplace | 105,888 |
| 18 | Worksite | 4,367 |
| 19 | Workstation | 37,513 |
| 20 | “Work location” | 683 |
| 21 | “Place of employment” | 562 |
| 22 | “Job site” | 1,115 |
| 23 | 14 or 15 or 16 or 17 or 18 or 19 or 20 or 21 or 22 | 157,219 |
| 24 | 6 and 13 and 23 | 3,403 |

All terms were searched in “title, abstract, keywords”.

PsycINFO search matrix

| PsycINFO | | |
| --- | --- | --- |
| 1: Implementation | 2: Physical activity | 3: Workplaces |
| Implement*  Execution  Compliance  Compliance [Th]  Adherence  Adjustment  Adjustment [Th] | “Physical activity”  Physical activity [Th]  “Physical exercise”  “Physical training”  Exercise  “Workplace intervention”  Workplace intervention [Th]  “Occupational health”  Occupational health [Th] | “Work place”  “Work site”  “Work station”  Workplace  Worksite  Workstation  “Work location”  “Place of employment”  “Job site”  Working space [Th]  “Working space” |

Th=Thesaurus

PsycINFO search protocol

| PsycINFO | | Date: 20/3 2018 |
| --- | --- | --- |
| **Nr. #** | **Search terms** | **Number of hits** |
| 1 | Implement* | 152,221 |
| 2 | Execution | 10,799 |
| 3 | Compliance | 33,139 |
| 4 | Compliance [Th] | 4,089 |
| 5 | Adherence | 25,429 |
| 6 | Adjustment | 99,213 |
| 7 | Adjustment [Th] | 15,716 |
| 8 | 1 or 2 or 3 or 4 or 5 or 6 or 7 | 303,522 |
| 9 | “Physical activity” | 32,301 |
| 10 | Physical activity [Th] | 16,447 |
| 11 | “Physical exercise” | 3,147 |
| 12 | “Physical training” | 683 |
| 13 | Exercise | 51,360 |
| 14 | “Workplace intervention” | 396 |
| 15 | Workplace intervention [Th] | 322 |
| 16 | “Occupational health” | 3,722 |
| 17 | Occupational health [Th] | 2,381 |
| 18 | 9 or 10 or 11 or 12 or 13 or 14 or 15 or 16 or 17 | 76,082 |
| 19 | “Work place” | 1,199 |
| 20 | “Work site” | 357 |
| 21 | “Work station” | 79 |
| 22 | Workplace | 32,614 |
| 23 | Worksite | 1,422 |
| 24 | Workstation | 466 |
| 25 | “Work location” | 88 |
| 26 | “Place of employment” | 131 |
| 27 | “Job site” | 75 |
| 28 | Working space [Th] | 323 |
| 29 | “Working space” | 369 |
| 30 | 19 or 20 or 21 or 22 or 23 or 24 or 25 or 26 or 27 or 28 or 29 | 468,605 |
| 31 | 8 and 18 and 30 | 515 |

Where nothing is indicated, “All fields” was searched.
