# Supplementary material for: Implementation strategies, and barriers and facilitators for implementation of physical activity at work: a scoping review
Source: Chiropr Man Therap. 2019 Oct 9;27:48. doi: 10.1186/s12998-019-0268-5 (PMC6784342; doi:10.1186/s12998-019-0268-5)
Supplement: Supplementary file 2 — TDF coding manual. TDF domains, definitions and constructs used to code barriers and facilitators. (DOCX 18 kb) [file 12998_2019_268_MOESM2_ESM.docx]

Additional file 2 (.docx). TDF coding manual. TDF domains, definitions and constructs used to code barriers and facilitators.

| Domain | Definition | Constructs | Notes |
| --- | --- | --- | --- |
| Knowledge | An awareness of the existence of something | Knowledge (including knowledge of condition /scientific rationale)  Procedural knowledge  Knowledge of task environment |  |
| Skills | An ability or proficiency acquired through practice | Skills  Skills development  Competence  Ability  Interpersonal skills  Practice  Skill assessment |  |
| Social/professional role and Identity (SPRI) | A coherent set of behaviours and displayed personal qualities of an  individual in a social or work setting | Professional identity  Professional role  Social identity  Identity  Professional boundaries  Professional confidence  Group identity  Leadership  Organisational commitment |  |
| Beliefs about Capabilities | Acceptance of the truth, reality, or validity about an ability, talent, or  facility that a person can put to constructive use | Self-confidence  Perceived competence  Self-efficacy  Perceived behavioural control  Beliefs  Self-esteem  Empowerment  Professional confidence |  |
| Optimism | The confidence that things will happen for the best or that desired goals will be attained | Optimism  Pessimism  Unrealistic optimism  Identity |  |
| Beliefs about Consequences | Acceptance of the truth, reality, or validity about outcomes of a  behaviour in a given situation | Beliefs  Outcome expectancies  Characteristics of outcome expectancies  Anticipated regret  Consequents |  |
| Reinforcement | Increasing the probability of a  response by arranging a dependent relationship, or contingency,  between the response and a given stimulus | Rewards (proximal / distal, valued / not valued, probable / improbable)  Incentives  Punishment  Consequents  Reinforcement  Contingencies  Sanctions |  |
| Intentions | A conscious decision to perform a behaviour or a resolve to act in  a certain way | Stability of intentions  Stages of change model  Transtheoretical model and stages of change |  |
| Goals | Mental representations of outcomes or end states that an individual wants to achieve | Goals (distal / proximal)  Goal priority  Goal / target setting  Goals (autonomous / controlled)  Action planning  Implementation intention |  |
| Memory, Attention and Decision Processes (MADP) | The ability to retain information, focus selectively on aspects of the environment and choose between two or more alternatives | Memory  Attention  Attention control  Decision making  Cognitive overload / tiredness |  |
| Environmental Context and Resources (ECR) | Any circumstance of a person's situation or environment that  discourages or encourages the development of skills and  abilities, independence, social competence, and adaptive behaviour | Environmental stressors  Resources / material resources  Organisational culture /climate  Salient events / critical incidents  Person x environment interaction  Barriers and facilitators |  |
| Social influences | Those interpersonal processes that can cause individuals to  change their thoughts, feelings, or behaviours | Social pressure  Social norms  Group conformity  Social comparisons  Group norms  Social support  Power  Intergroup conflict  Alienation  Group identity  Modelling |  |
| Emotion | A complex reaction pattern, involving experiential, behavioural,  and physiological elements, by which the individual attempts to deal  with a personally significant matter or event | Fear  Anxiety  Affect  Stress  Depression  Positive / negative affect  Burn-out |  |
| Behavioural Regulation | Anything aimed at managing or changing objectively observed or measured actions | Self-monitoring  Breaking habit  Action planning |  |

The coding manual is guided by definitions from Cane et al. *Implementation Science* 2012, 7:37.
Notes were added to the coding manual, as the experience with the coding process enhanced, and functioned as an aid in coding of the identified barriers and facilitators.
