# Supplementary material for: Implementation strategies, and barriers and facilitators for implementation of physical activity at work: a scoping review
Source: Chiropr Man Therap. 2019 Oct 9;27:48. doi: 10.1186/s12998-019-0268-5 (PMC6784342; doi:10.1186/s12998-019-0268-5)
Supplement: Supplementary file 3 — Overview of the TDF coding. Total number of identified factors and results of TDF coding for the included studies. (DOCX 17 kb) [file 12998_2019_268_MOESM3_ESM.docx]

Additional file 3 (.docx). Overview of the TDF coding. Total number of identified factors and results of TDF coding for the included studies.

| Author, year, country | Total factors identified, Barriers / Facilitators (n) | TDF Barriers (n) | TDF Facilitators (n) |
| --- | --- | --- | --- |
| Taylor et al.  2013, USA | 2 / 3 | SPRI (1)  ECR (1)  Social influences (1) | Knowledge (2)  Skills (1)  SPRI (1)  Beliefs about capabilities (1)  Intentions (1)  Goals (1)  Social Influences (1)  Emotions (1) |
| Taylor et al.  2014, USA | 3 / 4 | SPRI (1)  ECR (1)  Social influences (2)  Emotion (1) | SPRI (1)  Optimism (1)  Goals (1)  ECR (2)  Social influences (1)  Emotion (1) |
| Tudor-Locke et al.  2014, USA | 12 / 5 | Skills (2)  Beliefs about capabilities (2) Beliefs about consequences (1)  ECR (11)  Social influences (1)  Emotion (2) | Goals (2)  MADP (1)  ECR (1)  Emotion (1)  Behavioural regulation (1) |
| Justesen et al.  2017, DK | 9 / 5 | Knowledge (3)  Skills (3)  SPRI (5)  Beliefs about consequences (1)  Intentions (2)  ECR (4)  Social influences (3) | SPRI (5)  ECR (1)  Social influences (4) |
| Kinnafik et al.  2018, UK | 7 / 7 | Skills (1)  Beliefs about capabilities (2)  ECR (5)  Social influences (1) Behavioural regulation (1) | Knowledge (1)  Beliefs about capabilities (2) Beliefs about consequences (1) Intentions (1)  ECR (2)  Emotion (2)  Social influences (1)  Behavioural regulation (3) |
| Lawton et al.  2014, UK | 8 / 5 | SPRI (1)  Beliefs about consequences (2)  Intentions (3)  ECR (4)  Social influences (2) | SPRI (1)  Beliefs about capabilities (1) Intentions (1)  ECR (2)  Social influences (3) |
| Andersen & Zebis  2014, DK | 3 / 6 | ECR (3) | Skills (1)  Beliefs about capabilities (3) ECR (5)  Social influences (1) Behavioural regulation (1) |
| Mayer et al.  2013, USA | 7 / 9 | Skills (1)  Beliefs about capabilities (1) Beliefs about consequences (1)  Intentions (2)  Goals (1)  ECR (3)  Social influences (1)  Emotions (1)  Behavioural regulation (1) | Beliefs about capabilities (1) Reinforcement (2)  Goals (2)  ECR (1)  Social influences (2)  Emotion (1) |
| Bredahl et al.  2014, DK | 6 / 8 | Knowledge (1)  Beliefs about capabilities (1) Beliefs about consequences (2)  Emotions (1)  ECR (2)  Social influences (2) | Knowledge (3)  Skills (2)  SPRI (1)  Goals (2)  Social influences (4)  ECR (2) |

TDF = Theoretical Domains Framework, SPRI = Social/professional role and Identity, ECR = Environmental Context and Resources, MADP = Memory, Attention and Decision Processes.
